# Fertility Management in Cystinosis: A Clinical Perspective

**DOI:** 10.1016/j.ekir.2023.10.030

**Published:** 2023-11-03

**Authors:** Craig B. Langman, Rowena B. Delos Santos, Cybele Ghossein, Andrea M. Atherton, Elena N. Levtchenko, Aude Servais

**Affiliations:** 1 Feinberg School of Medicine, Northwestern University, Chicago, Illinois, USA; 2 Washington University in St Louis, St Louis, Missouri, USA; 3 Horizon Therapeutics plc, Deerfield, Illinois, USA; 4 Amsterdam University Medical Centre, Amsterdam, The Netherlands; 5 Necker Enfants Malades University Hospital, APHP; Imagine Institute, Paris, France

**Keywords:** cystinosis, extrarenal manifestations, fertility, lysosomal storage disorder, nephropathic cystinosis, reproductive health

## Abstract

Cystinosis is a rare, inherited, lysosomal storage disorder characterized by the progressive accumulation of intralysosomal cystine and subsequent organ and tissue damage. The kidneys are the first and most severely impacted organ. Although cystinosis was once considered a fatal pediatric disease, patients with cystinosis are living well into adulthood with advances in medical care, including kidney transplant and early and continuous use of cysteamine therapy. This increase in life expectancy has revealed an extrarenal phenotype of cystinosis that emerges in adolescence and adulthood, affecting nearly all body systems, including the endocrine and reproductive systems. As individuals with cystinosis are planning for the future, reproductive health and fertility have become areas of increased focus. This narrative review aims to summarize the current understanding of reproductive health and fertility in patients with cystinosis and discuss practical considerations for monitoring and managing these complications.

Nephropathic cystinosis is a rare, progressive, multisystemic lysosomal storage disorder occurring in approximately 1 in 100,000 to 200,000 live births.^1^, ^2^, ^3^ Cystinosis is caused by autosomal recessive inheritance of variants in the *CTNS* gene, which encodes for cystinosin, a lysosomal cystine transporter.^2^^,^^4^ The absence of functional cystinosin causes cystine to continuously accumulate within cells, eventually forming crystals and leading to tissue and organ damage throughout the body.^2^^,^^4^

Infants with cystinosis generally appear healthy at birth, with normal birth weight and initial growth.^5^ In the most severe form of the disease, infantile nephropathic cystinosis, the kidneys are the first and most severely affected organ, with symptoms of Fanconi syndrome, growth failure, and rickets emerging within the first year of life.^5^ Corneal cystine crystal formation can be seen on slit-lamp eye examination in children beginning at 12 months of age.^5^ Cystinosis is diagnosed by white blood cell cystine level measurement, genetic testing, and/or slit-lamp eye examination.^2^^,^^4^ Without treatment, or with delayed diagnosis, the progressive nature of cystinosis leads to multiorgan damage and the potential to reach kidney failure by the end of the first decade of life.^5^, ^6^, ^7^ Cysteamine is currently the only available disease-modifying treatment that depletes intralysosomal cystine.^1^^,^^4^ Despite early and continuous treatment with cysteamine, patients eventually require kidney transplant.^7^ After transplant, cystine continues to accumulate in extrarenal organs, causing complications that necessitate the use of supportive therapies.^4^^,^^8^^,^^9^ Investigational therapies, such as gene therapy, are currently in development and may play a future role in the management of this disease.^1^

With the advent of cysteamine therapy and advances in medical care, patients with cystinosis are now living into their 50s and beyond.^9^ What was once considered a fatal pediatric disease, cystinosis has been transformed into a chronic, progressive disease with impacts on nearly all body systems, including the endocrine and reproductive systems.^10^ In addition to the direct effects of cystine accumulation on reproductive organs, complications such as stunted growth, delayed development, hypothyroidism, and impaired renal function also affect the reproductive health and fertility of individuals with cystinosis (Figure 1).^11^^,^^16^^,^^17^ Cystinosis has especially significant impacts on male reproductive health, typically leading to infertility due to primary hypogonadism and azoospermia.^12^ Females with cystinosis are usually fertile; however, special attention must be given to their renal function, medications, and overall health status during preconception and pregnancy.^18^

**Figure 1:**
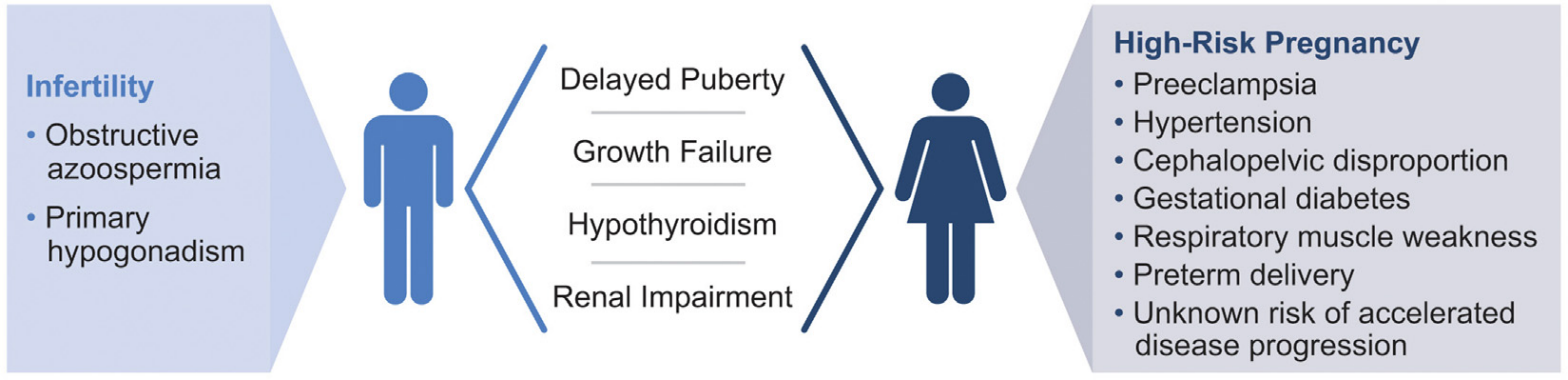
Impacts on the reproductive health of individuals with cystinosis.^1^^,^^11^, ^12^, ^13^, ^14^, ^15^ Delayed puberty, growth failure, hypothyroidism, and renal impairment affect the reproductive health in males and females with cystinosis. By adulthood, males are typically infertile, whereas females have normal fertility but are at high risk for pregnancy complications.

Given the importance and complexity of the topic, this review aims to summarize the current understanding of key mechanisms involved in the reproductive health and fertility of males and females with cystinosis and provide best practices to support the monitoring and management of these patients. In addition, we discuss the importance of routine conversations between the health care team and patients with cystinosis and their families about reproductive and sexual health, including fertility preservation options and family planning considerations.

### Reproductive Health and Fertility Management Considerations in Males With Cystinosis

Progressive reproductive complications ultimately lead to infertility in males with cystinosis. These patients commonly experience delayed puberty; low levels of testosterone; and increased levels of gonadotropins, luteinizing hormone (LH), and follicle-stimulating hormone.^12^^,^^16^ Kidney function and treatment with immunosuppressive agents appear to have little influence on the hormonal disturbances in males with cystinosis.^12^ Although primary hypogonadism was previously thought to be the prevailing mechanism of infertility, with the advent of cysteamine therapy, infertility in males with cystinosis has more recently been attributed to obstructive azoospermia and has been seen in patients with normal sex hormone levels (Table 1).^12^^,^^19^ In a mouse study, cysteamine was able to cross the blood-testis barrier but was unable to effectively reduce cystine accumulation in testicular tissue.^13^ Although cysteamine treatment does not negatively affect male fertility, it appears that good adherence to cysteamine therapy can only delay and not fully prevent testicular degeneration.^11^ These considerations underscore the importance of routine monitoring and discussions with males with cystinosis regarding development, reproductive health, and fertility as part of their multidisciplinary care.

**Table 1:** Proposed mechanisms of male infertility in cystinosis^11^^,^^12^

Primary Hypogonadism	Obstructive Azoospermia
Prevailing observations in males without early/continuous cysteamine therapy and in older patients	Hypothesized as the primary mechanism in the cysteamine era, observed starting in late adolescence
Evidence of crystal deposition, interstitial inflammation, and progressive atrophy of testicular tissue	Evidence of intratesticular crystalline buildup and damage, with rete testis, efferent ductules, and/or epididymal obstruction
Altered blood-testis barrier	Caput epididymis enlargement Dilation of rete testis
↑ LH, FSH ↓ Inhibin B (Sertoli cell dysfunction) ↓ Testosterone (Leydig cell dysfunction) ↓ Testicular volume over time	↓ Epididymal seminal plasma markers (NAG, ECM-1, fructose, zinc)

ECM-1, extracellular matrix protein-1; FSH, follicle-stimulating hormone; LH, luteinizing hormone; NAG, neutral alpha-glucosidase.

#### Prepubertal Males

Conversations about the reproductive health of males with cystinosis should begin at diagnosis. Following diagnosis, caregivers should receive counseling on the potential risk of infertility as part of the extrarenal phenotype of cystinosis. Growth should be evaluated regularly throughout childhood. If growth is stunted despite adequate nutrition, absence of rickets, correction of metabolic consequences of Fanconi syndrome, and effective cysteamine therapy, treatment with recombinant human growth hormone should be considered.^20^ Subcutaneous administration of recombinant human growth hormone (0.045–0.05 mg/kg/day) has been shown to improve growth without accelerating progression of chronic kidney disease (CKD).^20^^,^^21^

#### Pubertal Males

Delayed puberty is common in males with cystinosis and requires referral to pediatric endocrinology for further evaluation (Table 2). Delayed puberty is defined as having no signs of sexual maturation (testis volume <4 ml) until a chronological age that is 2.5 SDs above the mean age of puberty onset in a normal population, or 14 years of age for a Caucasian male.^23^ The onset of puberty may be delayed until 15 to 20 years of age in some male patients with cystinosis.^24^ Most males with cystinosis will spontaneously reach pubertal Tanner stages G4-5, P4-5, and A2 with a normal adult penile length; however, body hair typically remains sparse, and testes will not reach full adult volume in 20% to 80% of males.^11^^,^^12^^,^^19^

**Table 2:** Suggested endocrine and reproductive health evaluations and interventions for males with cystinosis

Type of Evaluation/Frequency	Endocrine/Reproductive Complications	Interventions for Consideration
**Prepubertal males (counseling topics: future reproductive health, infertility)**		
Growth evaluation, every 6 months	Delayed puberty^12^	Endocrinology referral^16^
Tanner stages, annually (>10 years old)		
Testis volume, annually (>10 years old)	Growth delays^16^^,^^20^^,^^21^	rhGH^16^^,^^20^^,^^21^
Bone age, annually (>10 years old)		
**Pubertal males (counseling topics: future reproductive health, infertility, fertility preservation options)**		
Growth evaluation, every 6 months	Growth delays^16^^,^^20^^,^^21^	rhGH^16^^,^^20^^,^^21^
Tanner stages, every 6 months Testis volume, every 6 months LH, FSH, testosterone, inhibin B, every 6 months Bone age, annually (until bone age of 18 years) Semen analysis, as indicated^11^^,^^12^^,^^18^	Delayed puberty^12^ Fertility preservation^18^	Fertility specialist, endocrinology, urology referrals^16^^,^^18^ Testosterone^18^^,^^22^ SERMs^22^ hCG^22^ Aromatase inhibitors^22^ Sperm cryopreservation ± TESE or PESA^11^^,^^12^
**Postpubertal males (counseling topics: infertility, fertility preservation options, family planning/genetic testing)**		
LH, FSH, testosterone, inhibin B, annually^18^ Semen analysis, as indicated^12^ Testis ultrasound, as indicated	Low testosterone^12^^,^^22^ Primary hypogonadism^12^ Azoospermia^12^	Endocrinology, urology referrals^16^^,^^18^ Testosterone^18^^,^^22^ SERMs^22^ hCG^22^ Aromatase inhibitors^22^ Fertility specialist, endocrinology, urology referrals^16^^,^^18^ Sperm cryopreservation ± TESE or PESA^11^^,^^12^ ARTs^11^

ART, assisted reproductive technique; FSH, follicle-stimulating hormone; hCG, human chorionic gonadotropin; LH, luteinizing hormone; PESA, percutaneous epididymal sperm aspiration; rhGH, recombinant human growth hormone; SERM, selective estrogen receptor modulator; TESE, testicular sperm extraction.

#### Postpubertal Males

Males with cystinosis commonly experience primary hypogonadism, characterized by low testosterone and inhibin B, indicating testicular Leydig and Sertoli cell dysfunction, respectively; and elevated gonadotropins, LH, and follicle-stimulating hormone.^12^^,^^24^ In a recent cross-sectional study of 18 males with cystinosis who were aged 15 to 40 years, elevated serum LH levels were observed only in males ≥20 years old, suggesting progressive Leydig cell dysfunction with age.^11^ Increased LH levels resulted in compensated Leydig cell dysfunction and normal serum testosterone levels; none of the males in this small cross-sectional cohort had low serum testosterone levels.^11^ Overall, normal sex hormones, including testosterone, inhibin B, LH, and follicle-stimulating hormone, have been observed in 50% to 90% of young adults who were adequately treated with cysteamine beginning early in life.^11^^,^^19^

Cystinosis is unique among conditions that cause male infertility; some publications have suggested that the primary pathology is defective sperm production, whereas others suggest coexisting obstructive and nonobstructive pathologies (Table 1).^12^^,^^24^ Sperm production is typically normal in obstructive azoospermia, but there is a blockage to the flow of sperm in the efferent ductules, epididymis, vas deferens, and/or ejaculatory duct of the prostate. Nonobstructive azoospermia is characterized by decreased or absent sperm production. With the introduction of early and continuous cysteamine therapy, evidence for the prevailing mechanism of male infertility in cystinosis has shifted from primary hypogonadism to obstructive azoospermia (Table 1).^11^^,^^12^^,^^24^ Testicular endocrine function may remain intact into the fourth decade of life with appropriate cystinosis management; however, azoospermia typically occurs starting in late adolescence.^11^

Two independent studies have pointed to the presence of obstructive azoospermia in male patients with cystinosis, characterized by enlarged caput epididymis; reduced epididymal seminal markers, including fructose, zinc, and extracellular matrix protein-1; and diminished seminal neutral alpha-glucosidase activity.^11^^,^^13^ Moreover, extensive crystalline buildup with lymphocyte or macrophage infiltration in the peripheral and central testicular tissues indicates the pathologic role of cystine accumulation and inflammation that lead to progressive testis damage in male patients with cystinosis.^13^ Alteration of the blood-testis barrier has been demonstrated in humans and cystinosis mice; however, it remains unclear whether this contributes to testis damage.^13^ Although libido and erectile function have not been studied systematically in males with cystinosis, the majority of examined patients have reported the ability to ejaculate.^19^^,^^24^

Given these findings, as soon as puberty is reached, males with cystinosis should have regular evaluation of sex hormone levels, semen analysis should be offered to determine the presence of azoospermia, and fertility preservation methods should be considered where appropriate (Table 2). Fertility preservation can be readily achieved in most male patients with cystinosis, including adolescents, through the cryopreservation of sperm. Surgical testicular or epididymal sperm extraction may be needed in patients who have developed azoospermia.

#### Testosterone Replacement Therapy in Males With Cystinosis

The American Urological Association guidelines on testosterone deficiency state that testosterone replacement therapy can be considered for patients with low serum testosterone levels if these low levels are accompanied by bothersome signs or symptoms, such as fatigue, decreased libido, erectile dysfunction, depressed mood, or anemia.^22^ Testosterone therapy may also benefit muscle health, which could be important for males with cystinosis.^25^ It can be administered in several forms, including topical gel, transdermal patch, intranasal spray, intramuscular injection, and subcutaneously implanted pellet.^22^ Many factors come into consideration when selecting a particular form of testosterone therapy, including dosing interval, insurance coverage, out-of-pocket cost, side effect profile, ease of use, and rates of compliance. Although exogenous testosterone therapy is generally well-tolerated, possible side effects include polycythemia, gynecomastia, acne, application or injection site irritation, and infertility. Infertility is a particularly important complication, because exogenous testosterone therapy can cause suppressed spermatogenesis and testicular atrophy.^26^ With long-term use, the suppressive effects of exogenous testosterone therapy on male reproductive potential can be permanent and irreversible.

The American Urological Association guidelines on testosterone deficiency also state that alternative therapies can be offered in place of exogenous testosterone in an effort to increase endogenous testosterone production and thus support rather than suppress spermatogenesis.^22^ These agents include selective estrogen receptor modulators, human chorionic gonadotropin, and aromatase inhibitors. Selective estrogen receptor modulators, such as clomiphene citrate, work by inhibiting estradiol negative feedback at the level of the hypothalamus and pituitary gland. Human chorionic gonadotropin is an injectable agent that acts as an LH agonist, thus stimulating Leydig cell production of testosterone. Aromatase inhibitors block the conversion of testosterone to estradiol, leading to increased serum testosterone levels through decreased estradiol negative feedback at the level of the hypothalamus and pituitary gland. These 3 classes of alternative therapies have different mechanisms of action; however, they collectively offer the same end effect in both stimulating endogenous testosterone production and supporting spermatogenesis.

#### Fertility Preservation, Sperm Harvesting, and Cryopreservation

Fertility preservation should be considered for any patient at risk of losing their reproductive potential.^27^ Given the high prevalence of azoospermia in males with cystinosis, more clinicians are advocating for semen analysis as early as possible after the onset of puberty (Table 2).^11^^,^^12^^,^^18^ If sperm are present in the semen sample, sperm cryopreservation can be pursued for fertility preservation. With cryopreservation, frozen sperm can be thawed in the future when patients desire conception and used for assisted reproductive techniques such as intrauterine insemination and *in vitro* fertilization. In patients with confirmed azoospermia, surgical testicular or epididymal sperm extraction can be attempted, and if sperm are found, they can also be cryopreserved for future use in assisted reproductive techniques. The first successful conception by a male with cystinosis was reported in 2018. Sperm were aspirated from the epididymis of a 27-year-old male and used for *in vitro* fertilization to achieve a pregnancy, which resulted in the birth of healthy fraternal twins.^28^ Patients are often concerned about the viability of cryopreserved sperm over time; however, this does not appear to be an issue as long as proper techniques are used for freezing and storage. Published reports describe successful paternity using sperm that have been cryopreserved for over 20 years in assisted reproductive techniques, including both intrauterine insemination and *in vitro* fertilization.^29^^,^^30^

Historically, many centers have shied away from offering fertility preservation to minor patients for reasons including provider discomfort, lack of access to reproductive specialists, concerns over obtaining assent and consent, and lack of access to fertility laboratories capable of processing semen specimens.^31^^,^^32^ Despite these barriers, the potential to find viable sperm in the semen before the occurrence of azoospermia suggests that semen analysis and sperm cryopreservation should be offered to males with cystinosis as soon as they reach puberty.^11^^,^^12^^,^^18^ Once a male has had nocturnal emission or ejaculation by self-stimulation, he is typically capable of providing a semen specimen for testing. Studies of cancer survivors who were not offered fertility preservation highlight the importance of discussing semen analysis and sperm cryopreservation as options with at-risk males. During qualitative focus group studies of adult survivors of pediatric cancer, males who were not offered fertility preservation expressed regret and disappointment in their medical providers.^33^ Many of these males had azoospermia as a result of their prior therapies; the missed opportunity for fertility preservation via sperm cryopreservation left many of these males unable to father biological children, causing substantial downstream emotional and interpersonal relationship impacts.

### Reproductive Health and Fertility Management Considerations in Females With Cystinosis

Females with cystinosis tend to have normal fertility despite delayed puberty, with menarche beginning around 15 to 19 years of age in historical cohorts, after which the protective effects of early cysteamine treatment have been demonstrated.^12^ Underlying CKD in females with cystinosis may also have a nonspecific impact on menarche and fertility.^34^ In addition, infertility is common in females with thyroid dysfunction, requiring treatment with L-thyroxine in cases of hypothyroidism.^35^ Female patients with cystinosis typically have normal menstruation and hormone levels if kidney function is preserved or after kidney transplant; however, they are considered at high risk of pregnancy-related complications due to the renal and extrarenal impacts of the disease. Complications related to cystinosis include preeclampsia, hypertension, gestational diabetes, hypothyroidism, muscle weakness, cephalopelvic disproportion related to short stature, and preterm delivery.^12^^,^^14^ In addition, there is an unknown risk of cystinosis disease progression during pregnancy.^12^^,^^15^

Females with cystinosis and CKD are at risk of severe antenatal and postnatal complications, particularly in the setting of advanced CKD or kidney failure. These complications include preeclampsia, CKD progression, and poor fetal outcomes, including intrauterine growth restriction, prematurity, low birth weight, and neonatal death.^14^^,^^15^ Females with cystinosis who have undergone kidney transplant are also at risk of gestational diabetes, pregnancy-induced hypertension, preeclampsia, miscarriage, stillbirth, and neonatal death, even with optimal allograft function.^36^, ^37^, ^38^ The frequency of preeclampsia in patients with cystinosis appears to be higher than in other females who have undergone kidney transplant.^14^ In addition, most females who received a transplant will undergo cesarean delivery rather than vaginal delivery.^36^, ^37^, ^38^ Risks to the children of mothers who received a transplant include preterm birth, low birthweight, and small for gestational age.^36^, ^37^, ^38^ Despite the early issues seen in children born to females who received a transplant, a cohort study reported that these children typically catch up in growth to term infants by 2 years of age.^36^ Finally, a small percentage of females can experience graft rejection during pregnancy, with allograft loss postpartum.^37^^,^^38^

Successful pregnancies have been described in females with cystinosis before and after kidney transplant.^14^^,^^15^^,^^39^ Before kidney transplant, pregnancy outcomes are dependent on renal function.^40^ After kidney transplant, it is recommended that females desiring pregnancy wait at least 1 year and have a stable immunosuppressive medication regimen, serum creatinine <1.4 mg/dL, minimal to no proteinuria, no history of graft rejection, and no ongoing infection.^38^^,^^41^ For some patients, waiting longer to become pregnant is recommended due to concern for the potential increased risk of all-cause graft loss and death-censored graft loss within the first 2 years posttransplant compared with after 2 years.^42^ Whether or not they underwent transplant, women with cystinosis should receive counseling on their fertility and contraceptive options. Some providers recommend that females with cystinosis concurrently use 2 different forms of contraception to prevent unplanned pregnancies. If a woman with cystinosis desires a pregnancy, it should ideally be planned with involvement from the multidisciplinary care team, including a nephrologist who is knowledgeable about the disease and a maternal-fetal medicine specialist. This is particularly important for females who have additional signs of systemic disease, including hypothyroidism, diabetes, hypertension, and pulmonary and neuromuscular difficulties, because these comorbidities can impact fertility and lead to poor pregnancy outcomes.

There are several considerations to discuss with patients before attempting conception. Steps should be taken to reduce the risk of preeclampsia and other complications during pregnancy. Given that females with cystinosis are at high risk of preeclampsia, a low dosage of acetylsalicylic acid should be considered for prevention.^14^^,^^43^ In addition, patients should be assessed regularly for risk of gestational diabetes, monitored for thyroid dysfunction, and evaluated for respiratory muscle weakness and the need for ventilator support peripartum and postpartum.^14^ Patients with diabetes should be managed with dietary changes and medications as appropriate. Medications, including cysteamine, antihypertensives, and immunosuppressives, must also be adjusted in women with cystinosis who desire pregnancy (Table 3). After delivery, patients should be counseled on restarting appropriate contraception.

**Table 3:** Recommended medication adjustments for females with cystinosis before, during, and after pregnancy^12^^,^^14^^,^^15^^,^^18^^,^^43^, ^44^, ^45^, ^46^, ^47^, ^48^, ^49^^,^^a^

Medication	Indication	Recommendation
**Preconception**		
Oral cysteamine	Cystine depletion	Continue until confirmed pregnancy
Acetylsalicylic acid	Prevention of preeclampsia	Consider adding to medication regimen due to high-risk status; can discuss on a case-by-case basis
Mycophenolate	Immunosuppression	Discontinue 6 weeks before attempts at conception; replace with azathioprine and monitor for tolerance
Belatacept and mTOR inhibitors (sirolimus, everolimus)	Immunosuppression	Consider change to calcineurin inhibitor; achieve consistent levels of medication in the blood and stable renal allograft function before attempts at conception
ACE inhibitors/ARBs	Antihypertensive	Replace with an alternative antihypertensive medication prior to attempts at conception
**Antepartum**		
Oral cysteamine	Cystine depletion	Recommend discontinuing use once pregnancy is confirmed; due to FDA pregnancy-risk category C,^b^ discuss potential for use on a case-by-case basis^c^
Azathioprine	Immunosuppression	Continue use through pregnancy
Calcineurin inhibitors (cyclosporine, tacrolimus)	Immunosuppression	Continue use through pregnancy; monitor medication levels in the blood and adjust dosage as needed to maintain therapeutic targets
Corticosteroids	Immunosuppression	Continue use through pregnancy
ACE inhibitors/ARBs	Antihypertensive	If a female becomes pregnant while taking these medications, discontinue use immediately and replace with an alternative antihypertensive medication
**Postpartum**		
Oral cysteamine	Cystine depletion	Restart as soon as feasible after birth if the mother does not plan to breastfeed^d^
Contraceptives	Prevention of pregnancy	Restart as appropriate
Calcineurin inhibitors (cyclosporine, tacrolimus)	Immunosuppression	Adjust medication dosage, likely to baseline, to maintain therapeutic targets

ACE, angiotensin-converting enzyme; ARB, angiotensin receptor blocker; FDA, US Food and Drug Administration; mTOR, mammalian target of rapamycin.

a After kidney transplant, it is recommended that females desiring pregnancy wait at least 1 year and have a stable immunosuppressive medication regimen, serum creatinine <1.4 mg/dL, minimal to no proteinuria, no history of graft rejection, and no ongoing infection.^38^^,^^41^^,^^42^

b Pregnancy-risk category C medication: risk cannot be ruled out; there are no satisfactory studies in pregnant women, and animal studies have demonstrated a risk to the fetus.^50^

c Consider consultation with maternal-fetal medicine specialist or genetic counselor.

d The decision to breastfeed should be assessed and discussed on a case-by-case basis.^44^

It is currently recommended that women with cystinosis discontinue cysteamine therapy as soon as pregnancy is confirmed due to potential teratogenicity.^12^^,^^15^^,^^18^ Cysteamine is considered a pregnancy-risk category C medication by the US Food and Drug Administration. The decision to continue a category C medication during pregnancy must come from discussion between a patient and her health care provider, with consideration for all benefits and risks. In some cases, the benefit of continuing therapy may outweigh the potential risk to the fetus. The use of cysteamine in pregnancy has only been reported in 1 case study; thus, the data are lacking.^51^ Cysteamine should be reinitiated as soon as feasible after birth, and particularly if the mother chooses not to breastfeed, to minimize time off therapy.^15^ The decision to breastfeed should be discussed between the patient and the health care team and should not be limited by the need to restart cysteamine therapy.^44^ Although insufficient data are available regarding the safety of cysteamine in breastfed infants, a single case report of a 24-year-old woman with cystinosis and preserved kidney function determined that the cysteamine concentration in breastmilk appears to be minimal.^44^ The patient received delayed-release cysteamine twice daily at a reduced dose (lowered from 825 mg to 675 mg twice daily).^44^ Cysteamine concentrations in breastmilk at 0, 2, 4, and 6 hours postdose were 0.12, 0.76, 1.87, and 0.51 mg/dL, respectively.^44^ Although infant cysteamine levels were not measured, they were predicted to be 0.4% of the dose, or 0.52 mg over a 24-hour period.^44^ Testing of the child at 18 months old indicated normal growth and development, with normal leukocyte cystine levels.^44^

In women who have undergone kidney transplant, choice and dosing of immunosuppressive medication will also require adjustments before or during pregnancy (Table 3). Immunosuppressive medications are generally continued throughout pregnancy, with the exception of mycophenolate. Mycophenolate, a teratogenic drug, should ideally be discontinued 6 weeks before attempts at conception and replaced with azathioprine.^15^^,^^18^^,^^45^ Patients should be monitored closely to ensure tolerance to azathioprine. Calcineurin inhibitors, such as cyclosporine and tacrolimus, can be continued during pregnancy; however, levels of these medications in the blood should be measured regularly throughout gestation. As the pregnancy advances, the blood levels of these medications tend to decrease due to increased CYP34A activity and reduced protein-binding, requiring dosage increases to maintain therapeutic targets.^46^ These medications will require adjustments again, likely to baseline dosages, during the postpartum period to keep levels in the therapeutic range.^46^ Despite an increased risk of gestational diabetes, individuals taking corticosteroids as part of their immunosuppressive regimen can continue taking these medications without dosage adjustments during pregnancy.^15^^,^^46^ Due to the limited safety information on belatacept and the mammalian target of rapamycin inhibitors, sirolimus and everolimus, patients may need to change to a calcineurin inhibitor and achieve consistent levels of medication in the blood and stable renal allograft function prior to attempts at conception.^15^^,^^46^

Females with cystinosis and high blood pressure should be counseled about the potential risks of angiotensin-converting enzyme inhibitors and angiotensin receptor blockers.^47^ These medications are contraindicated in pregnancy due to the risk of overall and cardiovascular congenital malformations, miscarriage, and stillbirth.^47^^,^^48^ Ideally, angiotensin-converting enzyme inhibitors and angiotensin receptor blockers should be replaced with an alternative antihypertensive medication prior to conception.^47^^,^^49^ If a pregnancy occurs, angiotensin-converting enzyme inhibitors and angiotensin receptor blockers should be discontinued immediately and replaced with an alternative option.^47^^,^^49^

Females with cystinosis should receive regular counseling on the likelihood of pregnancy, contraceptive options, and strategies to support a healthy pregnancy. Pregnancy is not recommended for patients with severely decreased estimated glomerular filtration rate. Close communication and coordination between the nephrology and obstetrics teams allow for preconception, antepartum, and postpartum medication changes; appropriate monitoring; and tailoring of care to patients’ kidney function and extrarenal complications.^18^

#### Fertility Preservation and Alternative Reproductive Options in Females With Cystinosis

Although females with cystinosis are typically fertile, they may receive counseling on fertility preservation and alternative reproductive options to reduce the risks associated with pregnancy in this disease. Fertility preservation is more involved in female than in male patients, but it is an option often delivered with success. The most common approach to fertility preservation in females is oocyte cryopreservation.^52^ Embryo cryopreservation is a less common option because many female patients undergoing fertility preservation might not yet have a chosen male partner for conception. Females with cystinosis might also choose to use an egg donor to avoid undergoing oocyte or embryo cryopreservation. Finally, in regions where it is permitted, females with cystinosis may choose to use a gestational surrogate to eliminate the risks associated with pregnancy and allow for continuation of cysteamine therapy.

### Reproductive Care Discussions, Coordination, and Unmet Needs

Considering that patients with cystinosis are surviving into adulthood, there is a growing need for health care teams to address both the transition to adult care and issues related to reproductive health. Nephrologists have long been the *de facto* primary care providers for patients with cystinosis, and with that comes the responsibility to support appropriate transition of care. Given the comprehensive and complex nature of caring for patients with cystinosis, a coordinated transition plan led by pediatric and adult nephrologists should be consistently implemented.

An effective transition from pediatric to adult care provides developmentally appropriate, uninterrupted health care aimed at maximizing the potential and functioning of transitioning patients.^53^ This transition period is often difficult for patients and their families. Patients who have received continuous care from trusted pediatric providers may experience anxiety during the transition due to changes in health care providers and differing approaches to care.^54^ This apprehension may also be shared by adult providers who are now tasked with caring for a rare and complex condition that has traditionally been viewed as a pediatric disease.

With the transition to adult care comes the need to address issues of personal autonomy and sexual and reproductive health. Health care providers often lack training and experience with addressing these sensitive issues, potentially leading to deficits in appropriate care. Research shows that adolescents and young adults with chronic illnesses share the same interests and concerns regarding sexual health as their healthy peers.^55^ Despite this, sexual and reproductive health is not consistently addressed during routine visits for adolescent and young adult patients, regardless of health status.^56^ A similar gap likely exists in the routine discussion of reproductive health and fertility in patients with cystinosis.

Although there are currently no specific guidelines on the management of reproductive health for adolescents and young adults with cystinosis, expert guidance on cystinosis management more generally highlights the importance of early counseling and involving relevant specialists, including fertility experts, endocrinologists, obstetrician-gynecologists, and urologists.^16^^,^^18^ Indeed, a team-based approach, including the primary care provider, nephrologist, and other multidisciplinary specialists, is ideal for comprehensively addressing the reproductive health of individuals with cystinosis. Conversations about reproductive health, fertility status, fertility preservation, contraception, and pregnancy should be initiated by the pediatric health care team and continued as soon as the patient transitions to adult care. Given their integral role in caring for patients with cystinosis, nephrologists should introduce these conversations and help coordinate referrals to other specialists.^18^

### Family Planning and Counseling

Integrating genetic counseling into the cystinosis multidisciplinary care model is recommended to improve the understanding of reproductive health options and family planning and to allow patients and their caregivers to make informed decisions in a timely manner.

All patients with cystinosis should be offered preconception genetic counseling to discuss options related to family planning. Because cystinosis is an autosomal recessive disorder, requiring the inheritance of 2 variants in the *CTNS* gene, individuals with cystinosis and their partners, especially consanguineous couples, could be offered genetic testing if it has not been previously completed.^57^ In cases of consanguineous marriage, genetic testing of the unaffected partner is recommended. The likelihood of offspring having the disease is very low, assuming the other partner is unaffected and is not a carrier of a *CTNS* gene variant (Figure 2). All children born to a parent with cystinosis will be obligate carriers (Figure 2a). If both partners have cystinosis, each pregnancy will result in the child having the disease. If 1 partner has cystinosis and the other partner is a carrier, there is a 50% chance that each pregnancy will result in the child having cystinosis (Figure 2b). It is recommended that the unaffected partner be offered carrier testing. Although molecular testing can identify *CTNS* variant(s) in 95% of individuals with cystinosis, there is still a possibility that the individual possesses a variant that has not yet been identified or is difficult to detect with current technologies.^5^^,^^12^^,^^57^

**Figure 2:**
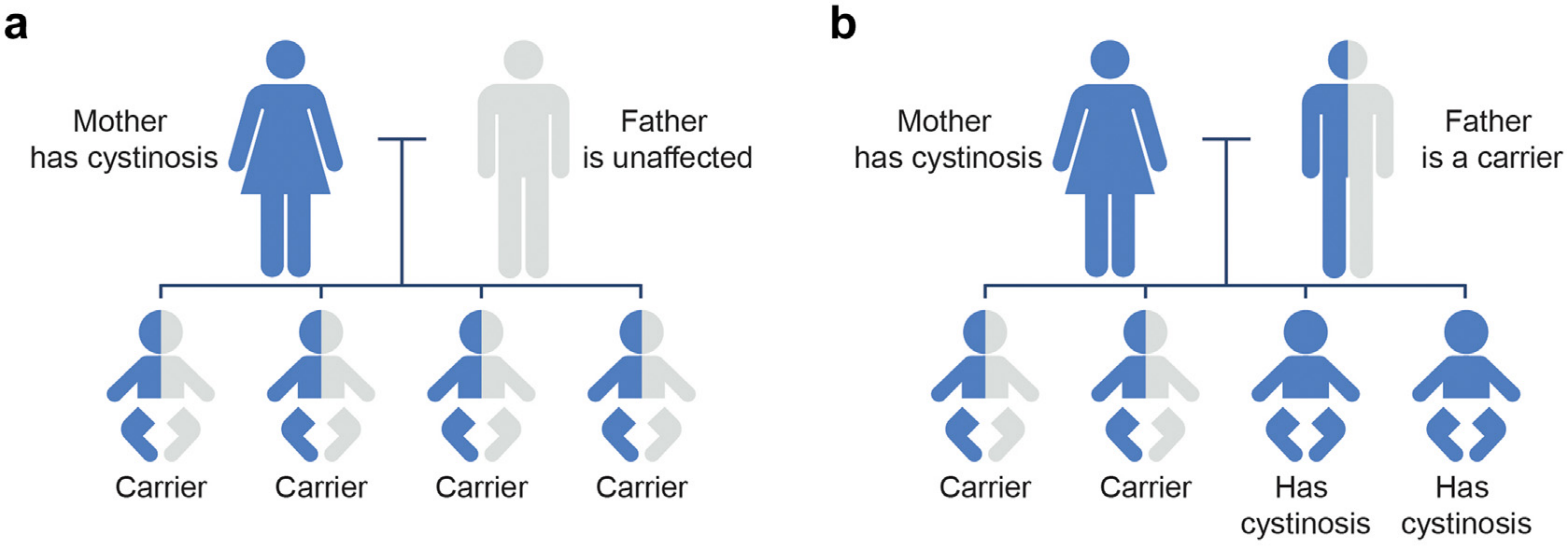
Examples of autosomal recessive inheritance patterns in cystinosis. (a) Risk of inheritance if the mother has cystinosis and the father is unaffected. All children will be obligate carriers and will not be affected by the condition.^57^ (b) Risk of inheritance if the mother is affected and the father is a carrier. With each pregnancy, there is a 50% chance that the child will have cystinosis and a 50% chance that the child will be a carrier of the condition.^57^

During preconception genetic counseling, patients with cystinosis and their families will discuss recurrence risks, learn about available genetic screening and testing options, and receive education about possible reproductive options. Throughout these counseling sessions, it is important to note that the risk of an individual with cystinosis having an affected child is very low if their nonconsanguineous partner is unaffected and does not carry an identifiable *CTNS* variant*.* Prenatal diagnostic testing via chorionic villus sampling and amniocentesis is an option for couples at an increased risk of having a child with cystinosis; however, the potential benefit may not outweigh the risk of miscarriage and bleeding.^57^^,^^58^ Instead, the newborn can be tested for cystinosis using cord blood or genetic testing at the time of birth.

### Summary

Patients with cystinosis experience significant impacts to their reproductive health and fertility as part of the extrarenal phenotype of the disease. Males with cystinosis are typically infertile as a result of hypogonadism, impaired spermatogenesis, and obstructive azoospermia. From diagnosis, males with cystinosis and their families should be counseled on the risk of infertility, potential options for fertility preservation, and use of assisted reproductive techniques in order to father biological children. Successful pregnancies have been reported in females with cystinosis; however, special care must be given to these patients due to polypharmacy and comorbidities, such as CKD, hypothyroidism, hypertension, diabetes, and pulmonary and neuromuscular complications. Key themes highlighted in this narrative review include the importance of multidisciplinary care team involvement, tailored management, and routine counseling of patients and their families on these essential topics. As the natural history of cystinosis continues to evolve, patients and their caregivers are looking to visualize a future beyond their diagnosis, a considerable motivator in maintaining patient care engagement.^59^ Across disciplines and beginning in childhood, clinicians can help instill and preserve hope for the future in patients with cystinosis and their families through candid conversations about fertility status and proactive discussions about family planning.

## Disclosure

CBL, RBDS, and CG have received honoraria from Horizon Therapeutics plc for consulting/advisory activities; AMA is an employee of and owns stock in Horizon. ENL and AS have received honoraria from Recordati Rare Diseases S.p.A. and Chiesi Farmaceutici S.p.A. for consulting/speaking activities. ENL has received honoraria from Kyowa Kirin Co., Ltd. for consulting activities. CBL has received honoraria from Eli Lilly and Company and Dicerna Pharmaceuticals/Novo Nordisk A/S for consulting activities. RBDS has received honoraria from Veloxis Pharmaceuticals, Inc, and Alexion Pharmaceuticals for consulting/advisory activities; as part of her employment at Washington University in St Louis, she has received grant funding from Merk & Co Inc, Novartis AG, Veloxis Pharmaceuticals Inc, and CareDx Inc. RBDS has received royalties from UpToDate.
